# Acceptance of a mobile telepresence robot used to teach adapted physical activity to isolated older adults: extending and testing the technology acceptance model

**DOI:** 10.3389/fpubh.2024.1405231

**Published:** 2024-07-09

**Authors:** Elodie Navarro, Jean-Jacques Temprado, Nicolas Mascret

**Affiliations:** Aix Marseille Univ, CNRS, ISM, Marseille, France

**Keywords:** technology acceptance, mobile telepresence robot (MTR), physical activity, older adults, videoconference, teaching

## Abstract

This study aimed to investigate the acceptance of adapted physical activity (APA) by teachers and students before the use of a mobile telepresence robot (MTR), used to remotely supervise isolated older adults’ physical activity. While previous studies have shown MTR to be fairly well accepted by older adults, nothing is known about its acceptance by APA teachers themselves. However, if they did not accept it, the MTR would not be used in the end. This would be a public health issue because isolated older adults would not benefit from supervised APA, yet beneficial to their health. To this end, 334 participants answered a survey that measured different psychological variables, based on the technology acceptance model (TAM). Student’s *t-tests* and structural equation modeling were used for data processing. Results showed that, before use, there was not any significant difference between teachers’ and students’ acceptance of the MTR. Then, perceived usefulness for teaching APA, perceived ease of use, perceived enjoyment, and intention to use the MTR were lower than the mean of the scale, while perceived usefulness for older adults was higher than the mean of the scale. Finally, this study has validated an extended version of the TAM (including the need for competence and MTR self-efficacy), which allowed it to explain 84.3% of the variance of the students’ and APA teachers’ intention to use the MTR for teaching APA to isolated older adults. Initial obstacles to the use of the MTR seem to exist on the part of APA teachers, prior to their first use, whereas this is not the case for older adults. APA teachers’ acceptance should therefore be investigated in future studies to examine whether this trend is confirmed after the effective use of the MTR.

## 1 Introduction

Enhancing successful aging and wellbeing of the growing number of older adults in the general population is a public health challenge for science and society (1, 2). Most studies have demonstrated that regular physical activity (PA) is both an effective and inexpensive way to achieve this objective (3–6).

However, although 9 of 10 older people know the benefits of PA on healthy aging (7), most of them are sedentary: 67% of older adults stay sitting more than 8.5 h a day (8), and according to Trost et al. (9), as age increases, the level of daily PA decreases. Among the factors that may explain these observations, an important one relates to the possibility of accessing PA programs supervised by qualified professionals for isolated older adults, for those who are living far from structures that offer PA, or even for those who would prefer to practice at home but do not find any satisfying solution to do it (9, 10). To overcome this problem, several options, based on technologies, are currently available: (i) using (at home) autonomous robots fitted with artificial intelligence [e.g., (11, 12)], (ii) attending to remote sessions provided by videoconference [e.g., (13, 14)], or (iii) using a web platform that offers videos recorded by specialized coaches^1^^,^^2^.

Autonomous (fully automated) robots may be used for the animation of PA sessions by mimicking the requested movements, but they lack inter-personal interactions, neither providing instructions and feedback to the participants nor allowing them to adapt the exercises to each participant. The same criticism applies to most of the platforms that offer videos. On the other hand, videoconference, which, since COVID, is a growing trend for teaching PA remotely by professionals, allows interaction with the PA teacher. However, due to the constraints imposed by the fixed video system, exercises involving body movements of large amplitude and displacements are impossible as individuals exit from the teacher’s field of vision. Moreover, when the participant moves away from the computer or tablet, it becomes difficult for the videoconferencing teacher to see him or her accurately, as the participant appears very small on the screen. Thus, on the one hand, several functionally important exercises (i.e., those involving mobility) cannot be practiced and, on the other hand, it is more difficult, if not impossible, for the PA teacher to provide accurate instructions and feedback to the participants, which are critical for safety and progress (15).

**Figure 1:**
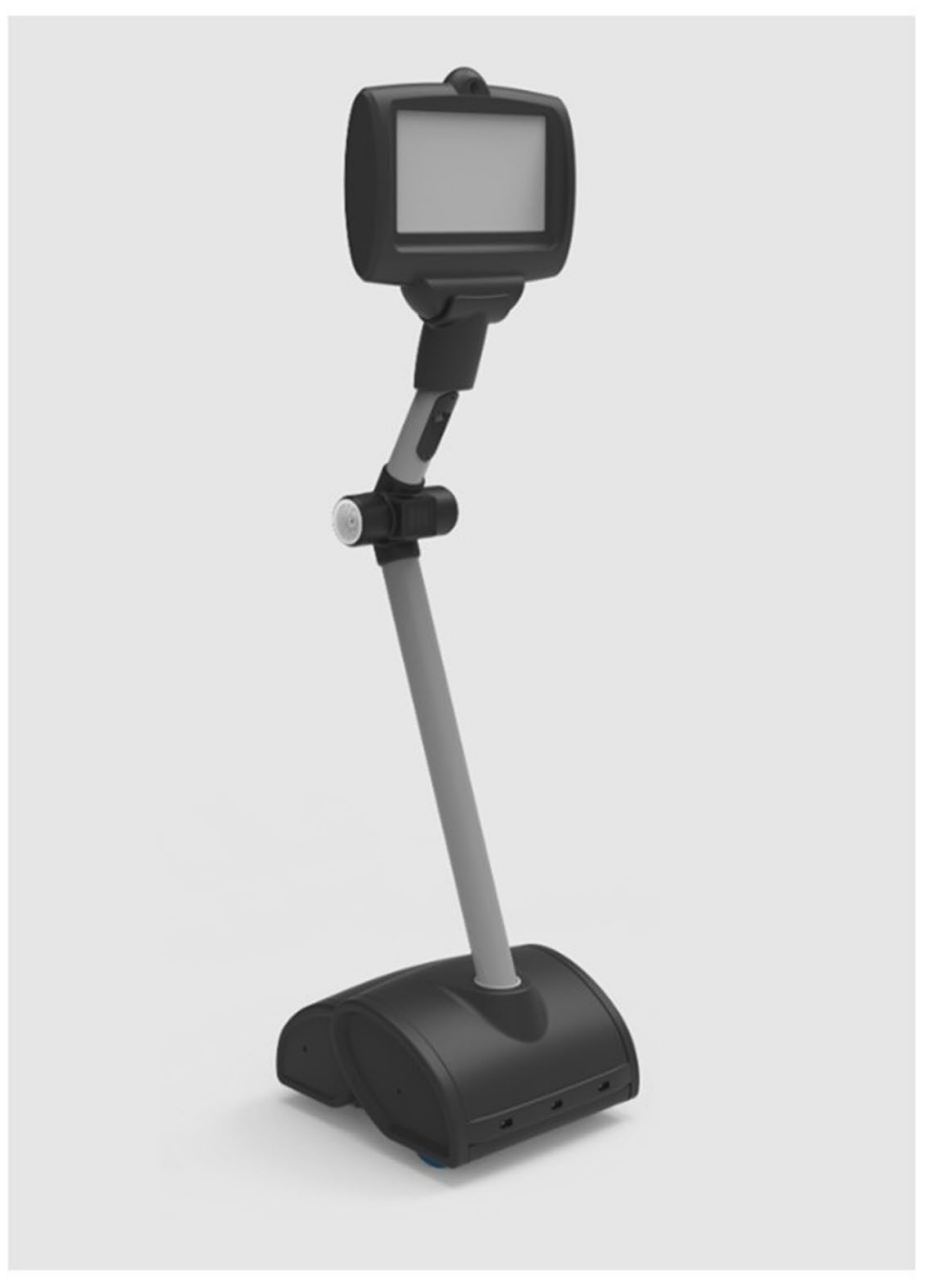
Picture of the Ubbo mobile telepresence robot. Reprinted with permission from Axyn Robotique, © Axyn Robotique.

Mobile telepresence robot (MTR) may help to overcome these limitations for teaching PA remotely. MTR looks like a videoconference system mounted on a mobile base, thereby allowing the pilot to move the robot to better interact with the participants. Thus, relative to videoconference, MTR has added value as it allows the operator interacting via two-way video and audio on a mobile base (16). However, this type of device has been rarely used to supervise PA remotely. The present study capitalizes on the use of such an MTR namely *Ubbo* (*Axyn Robotique^™^*) to test its acceptance by PA teachers. *Ubbo* is 1.60 m tall, and it allows the pilot to move in a remote environment, to hear and to be heard in real time due to its speakers and its microphone, and to see and to be seen due to its screen and its two cameras. It is controlled through an online interface available on a computer, laptop, digital tablet, or smartphone, from anywhere in the world, the only constraint being having access to a Wi-Fi or 4G network (Figure 1).

Presumably, using *Ubbo* to supervise adapted physical activity (APA) might be appropriate to provide individualized instructions and feedback to participants, by bringing the MTR close to them during the course of exercises due to its displacement and its moveable head (17). Thus, as MTR needs APA teachers to operate and as it capitalizes on their expertise to be effective, it should be well accepted by this population. It is not that simple, however, as technology acceptance does not only depend on the objective effectiveness of technology (18).

The term “acceptability” refers to acceptance of a technology before use (19). In the present study, we will use “acceptance before use” instead, which is the most widely used term in the literature. According to Mascret et al. (20), studying acceptance of technology before use is relevant because (i) individuals have an opinion on a technology even if they do not have used it, based on their own representations, (ii) the roll-out of a technology depends on its acceptance before use (a technology that has little or no acceptance before its first use is likely to be less widely used thereafter), and (iii) it highlights that some psychological antecedents could promote, block, or threaten the intention to use it. Of course, this initial opinion may evolve after an effective use of the technology, or it may be maintained (19). However, studying acceptance of technology before a first use allows to highlight factors and contexts allowing a better adaptation of technology devices (21). This provides a prediction about the way a technology should be integrated (or not) in a particular professional context. Thus, we chose in the present study to examine MTR acceptance before use for investigating this issue, as a necessary first step in understanding the acceptance process (22, 23).

The most frequently used model to study acceptance of technology is the technology acceptance model (TAM) (22–24). It conceptualizes the positive relationship between three variables: perceived usefulness, perceived ease of use, and intention to use. According to Davis (24), perceived usefulness refers to “the degree to which a person believes that using a particular system would enhance his or her job performance.” Perceived ease of use is defined as “the degree to which a person believes that using a particular system would be free of effort” [(24), p. 320]. A strong prediction of the TAM is that the more someone thinks that the technological device is useful and easy to use, the more he/she has the intention to use it and the more he/she uses it effectively. In the present study, the effective use of the MTR *Ubbo* was not considered because MTR acceptance was investigated only before its first use. Perceived enjoyment was then added to the TAM, which is “the extent to which the activity of using the device is perceived to be enjoyable in its own right, apart from any performance consequences that may be anticipated” [(25), p. 1113]. In many studies, perceived enjoyment significantly and positively influenced the intention to use technology. In the present study, the TAM was used with APA teachers to examine their acceptance before the use of an MTR (*Ubbo*) intended to teach APA remotely to isolated older adults.

Only three studies have investigated the acceptance of MTR intended to supervise older adults’ PA. Mitzner et al. (26) investigated the acceptance by older adults with mobility impairments of an MTR used for PA, using questionnaires and interviews. Results showed that the 14 participants were quite prone to accept MTR, under the reserve that they identified benefits, including usefulness and ease of use. Another study investigated specifically acceptance by 230 older adults, before use, of the MTR *Ubbo* for supervising PA (15). Results showed that the MTR *Ubbo* was significantly, but lowly, found by older adults to be useful, easy, and pleasant to use. However, older adults had no significant intention of using it, but they did not refuse it either. In the same way, Mascret, Vors and Temprado (17) studied MTR acceptance by older adults after a first use. They found that participants thought MTR was significantly useful, easy to use, and enjoyable to use, and they intended to use it for PA. As these previous studies focused on older adults only, the question remains however of whether the MTR is also accepted by APA teachers. If older adults accept the MTR but their APA teachers do not, it is highly likely that the MTR will not be used despite its potential benefits for supervising APA remotely, which would not change the public health issue linked to the lack of supervised APA among isolated older adults.

To address this issue, the TAM needs to be adapted. Two adaptations of the model have been conducted. First, based on the distinction highlighted by Mayer and Girwidz (27) for teachers in the academic domain, two aspects of perceived usefulness need to be distinguished: perceived usefulness of the MTR for teaching APA to isolated older adults remotely (i.e., the MTR may improve the APA sessions themselves, for example, by improving their organization or their individualization) and perceived usefulness of the MTR for keeping or improving isolated older adult’s physical skills (i.e., the MTR may maintain or enhance isolated older adults’ performance).

Second, external variables have been added to the TAM to improve its explanatory power (24). The first variable is “robot self-efficacy,” that is, beliefs that an individual has about its own capabilities to mobilize the motivation, cognitive resources, and courses of action necessary for the MTR use (28, 29). Robot self-efficacy was added to the tested model as it influenced perceived ease of use (30) and was positively correlated with acceptance (31). “Need for competence,” that is, “individuals’ inherent desire to feel effective in interacting with the environment” [(32), p. 982], was also added to the model. It is one of the three psychological needs that are fundamental determinants of behavior (33). Moreover, the need for competence significantly and positively predicted both perceived usefulness and perceived ease of use (34). Need for competence focused on the potential effectiveness as an APA teacher, while robot self-efficacy focused specifically on the potential effectiveness with the MTR. In summary, the APA teachers’ belief about their capacity to use MTR and their concern about their need for improved teaching skills could influence their MTR perceived ease of use.

Finally, few studies reported a positive evolution of technology acceptance with work experience [e.g., (35)], while some other studies showed that experienced and non-experienced teachers in the academic domain had similar intentions to use a technology [e.g., (36)]. Moreover, a resistance to change phenomenon (37) may occur among experienced teachers when they must include something new in their usual professional activity, particularly when it involves including a new technology with which they are unfamiliar. In contrast, inexperienced teachers currently in training may be considered digital natives, regularly using technologies in their daily lives and studies (38). Consequently, they were more likely to use technologies in their APA sessions than more experienced APA teachers, which could lead to a higher level of MTR acceptance. Consequently, the effect of professional experience on MTR acceptance was also tested in the present study, by comparing MTR acceptance of APA students and APA teachers.

### 1.1 The present study had two main objectives

The first one was to test the validity of the TAM, extended with (i) robot self-efficacy and need for competence and (ii) perceived usefulness divided into two parts: perceived usefulness for teaching APA to isolated older adults and perceived usefulness for keeping or improving older adults’ physical skills. Indeed, validating the TAM would explain the determining factors of APA teachers’ intention to use the MTR, before a first use, to remotely supervise isolated older adults’ APA sessions.

**Figure 2:**
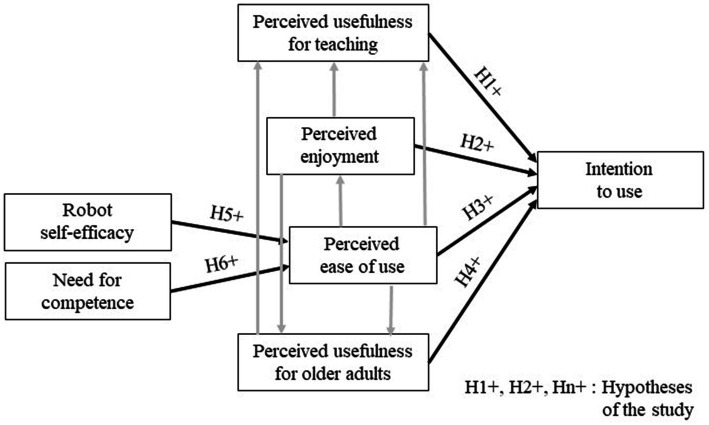
Model tested in the present study. The grey arrows correspond to the predictions that must be tested according to the TAM procedure but do not represent the main hypotheses of the present study.

Six hypotheses were tested, represented in Figure 2: APA teachers’ and students’ intention to use the MTR should be positively predicted by: perceived usefulness for teaching APA to isolated older adults (H1+), perceived enjoyment (H2+), perceived ease of use (H3+), and perceived usefulness for keeping or improving isolated older adult’s physical skills (H4+). Concerning external variables, perceived ease of use should be positively predicted by robot self-efficacy (H5+) and need for competence (H6+).

The second objective of the present study was to determine the influence of professional experience by comparing MTR acceptance of APA students and APA teachers. First, based on the potential benefits of the MTR *Ubbo* to remotely supervise APA for isolated older adults, we hypothesized that perceived usefulness for teaching APA to isolated older adults, perceived usefulness for keeping or improving isolated older adult’s physical skills, perceived enjoyment, and intention to use the MTR would be significantly higher than the mean of the Likert scale for both APA students and APA teachers (H7). Second, we hypothesized that perceived ease of use would be lower than the mean of the Likert scale both for APA students and APA teachers (H8) because remotely controlling the MTR to supervise older adults’ APA has never been done by the participants previously. Third, we hypothesized that the scores of all the TAM variables would be higher for APA students than for APA teachers (H9) because APA students are digital natives and more used to include technologies in APA sessions than experienced APA teachers.

## 2 Methods

### 2.1 Participants and procedure

This study included 334 French volunteers (121 men and 213 women) aged between 19 and 62 (*M*_age_ = 24.90 years, SD = 7.00). This sample was divided into two groups. First, the students’ group was composed of 195 participants (70 men and 125 women, aged between 19 and 53, *M*_age_ = 20.82 years, *SD* = 2.96), who were students between second and fifth years in the APA university diploma, which are higher studies giving the APA teacher’s grade. The second sample was composed of 139 APA teachers (51 men and 88 women, aged between 21 and 62, *M*_age_ = 30.63 years, *SD* = 7.02) with professional experience (*M* = 6.29 years, SD = 5.88). To be included in the present study, the only eligibility criteria that participants had to follow was to be either a student in the APA sector to take part in the students’ group, or a professional APA teacher (employed in a structure or self-employed) to take part in the professionals’ group.

Data were collected online through a free and secured software named *Framaforms*. In order to recruit participants, researchers intervene with APA students in the surrounding universities, and with APA teachers in the surrounding structures, transferring the link to the online survey. They also diffused the link via e-mail to all the universities of France offering APA courses of studies, and to organizations proposing APA. The first page of the questionnaire was a text presenting the MTR *Ubbo*, accompanied by a picture (because most people have never seen a MTR and do not know what it looks like). Its functionalities and its potential use to teach APA to isolated older adults were highlighted in the text (Appendix A). Participants had to read this presentation and then reply to a questionnaire assessing the variables of the TAM and demographic information (i.e., gender, age, frequency of social robot use, and frequency of APA teaching to older adults), which is a usual procedure in studies conducted with the TAM to investigate the acceptance of a device by older adults before a first use [e.g., (39, 40)]. The answers were anonymous, and data protection was provided by the *Framaforms* software. The National Ethics Committee approved the present study (ref. IRB00012476-2023-25-05-253), and participants were free to withdraw from the study at any time without the need for justification. A video illustrating the MTR used in an APA context is available in Supplementary material; nevertheless, it was not presented to the participants.

### 2.2 Measures

#### 2.2.1 MTR acceptance

Based on several studies (40–44), the first survey included 15 items about the 5 variables of the TAM translated in French and adapted to MTR that participants scored on a Likert scale from 1 (*strongly disagree*) to 5 (*strongly agree*) (Appendix A). Participants responded to three items assessing perceived usefulness for teaching APA to isolated older adults (e.g., *“*I *think that MTR would be useful to teach remotely APA to isolated older adults”*), three items assessing perceived usefulness for keeping or improving older adults’ physical skills (e.g., *“Using MTR to teach APA would allow keep or improve isolated older adults’ physical skills”*), three items assessing perceived enjoyment (e.g., *“Using MTR to teach remotely APA to isolated older adults would be enjoyable”*), three items assessing perceived ease of use (e.g., *“I think that using MTR to teach remotely APA to isolated older adults would be easy”*), and three items assessing intention to use (e.g., *“If I had the opportunity to have access to this MTR, I probably will use it to teach remotely APA to isolated older adults”*). Internal consistency was verified by McDonalds’ omegas (45), which were acceptable for all variables (ranging from 0.748 to 0.935).

#### 2.2.2 External variables

The second survey included three items assessing robot self-efficacy [e.g., *“I am confident in my ability to use the MTR”*; (29)] and four items assessing APA teachers’ need for competence [e.g., *“In my APA teacher job, I feel that I can do even the most difficult tasks”*; (32)] (Appendix A). These items were scored by the participants on a Likert scale from 1 (*strongly disagree*) to 5 (*strongly agree*), and their internal consistency was also checked using McDonalds’ omega. It was acceptable for the need for a competence variable (ω = 0.850). However, it was quite low for the MTR self-efficacy, but close to the reference value of 0.700 (ω = 0.669). Hence, statistical analyses were still carried out, but the results of the need for competence were analyzed with caution.

#### 2.2.3 General information

General information about participants was asked in the last survey to characterize the population and to assess control variables: gender, age, frequency of social robot use, and frequency of APA teaching to older adults.

### 2.3 Data analyses

Data analyses were conducted using the JASP software (version 0.16.2.0), and the significance threshold was considered at *p* < 0.05. First, potential outliers were detected by using the Mahalanobis distance. Second, the data normality of each variable was tested with skewness value ≤ |2| and Kurtosis value ≤ |7| (46). Then, the homogeneity of each participant’s group variance was checked for each variable by a Levene test. Thus, parametric statistical tests were allowed on these data. A confirmatory factor analysis (CFA) was used, and fit indices were calculated to evaluate the model fit (47, 48): the *χ2/df* ratio (a value ≤3 is needed), the CFI (Comparative Fit Index; a value ≥0.90 is needed), the TLI (Tucker–Lewis Index; a value ≥0.90 is needed), the RMSEA (root mean square error of approximation; a value ≤0.08 is needed), and the SRMR (standardized root mean square residual; a value ≤0.08 is needed). Then, a structural equation modeling (SEM) analysis was carried out to test the hypotheses. Control variables were entered in the model: gender, age, frequency of social robot use, and frequency of APA teaching to older adults. However, for achieving maximum parsimony, if a control variable was not found to be a significant predictor of one of the model variables, it was deleted (49).

One sample *t*-tests were then conducted for each variable (perceived usefulness for teaching APA to isolated older adults, perceived usefulness for keeping or improving older adults’ physical skills, perceived ease of use, perceived enjoyment, and intention to use) to compare the scores with the mean of the Likert scale (i.e., 3), both for APA students and APA teachers. Finally, *t*-tests for independent samples were conducted to examine potential differences for each TAM variable between APA students and APA teachers.

## 3 Results

### 3.1 Preliminary results

Mahalanobis distance revealed that one participant was an outlier, who had been removed from the final sample. Data normality was also checked for each group, by calculation of skewness (max = |0.623|) and kurtosis (max = |0.882|) coefficients (Table 1). Then, a Levene test was performed to check the variance homogeneity of the two groups, which has been confirmed (*p* = 0.716).

**Table 1:** Descriptive statistics, skewness, kurtosis, and McDonald’s omegas.

	Mean	Standard deviation	Skewness	Kurtosis	McDonald’s omegas
Perceived usefulness for teaching	2.701	0.939	0.019	−0.670	0.801
Perceived usefulness for older adults	3.416	1.031	−0.399	−0.560	0.920
Perceived ease of use	2.617	0.916	0.307	−0.301	0.748
Perceived enjoyment	2.554	0.943	0.059	−0.768	0.836
Intention to use	2.383	1.151	0.405	−0.875	0.935
Robot self-efficacy	3.001	0.927	0.129	−0.341	0.669
Need for competence	3.919	0.678	−0.611	0.464	0.850
Age	24.901	6.995	2.047	5.144	-

### 3.2 Validation of the TAM

**Figure 3:**
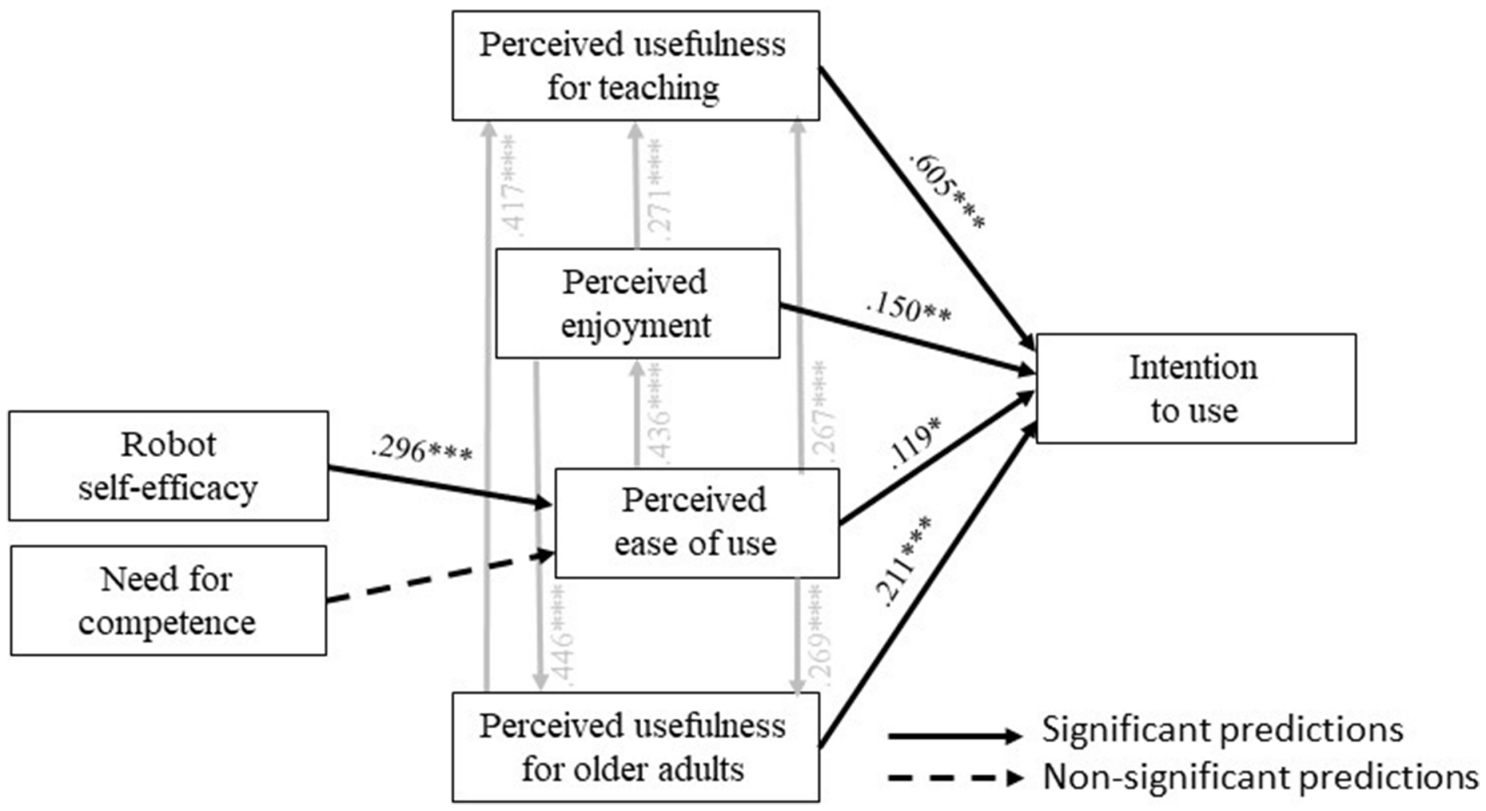
Validated structural model with standardized path coefficients. *p < 0.05, **p < 0.01. *** *p* < 0.001. The grey arrows correspond to the predictions that must be tested according to the TAM procedure but do not represent the main hypotheses of the present study.

First, the results of the CFA conducted on the covariance matrix of the TAM items confirmed the hypothesized seven-factor model (*χ*^2^(188, *N* = 334) = 350.141, *p* < 0.001, *χ*^2^/df = 1.862, CFI = 0.962, TLI = 0.954, RMSEA = 0.051, SRMR = 0.059). Then, the SEM analysis showed that the model fit the expected requirements (*χ*^2^(8, *N* = 334) = 20.947, *p* = 0.05, *χ*2/df = 2.618, CFI = 0.985, TLI = 0.963, RMSEA = 0.070, SRMR = 0.040). Figure 3 highlights that participants’ intention to use the MTR *Ubbo* was positively predicted by perceived usefulness for teaching APA (*p* < 0.001), perceived usefulness for improving older adults’ skills (*p* < 0.001), perceived enjoyment (*p* = 0.005), and perceived ease of use (*p* = 0.023). These findings mean that the more the APA teachers think that the MTR *Ubbo* is useful, enjoyable to use, and easy to use, the more they have the intention to use it to teach APA to isolated older adults. Consequently, H1, H2, H3, and H4 were validated. Furthermore, perceived ease of use was positively predicted by robot self-efficacy (*p* < 0.001). In other terms, the more the participants were confident in their capability to use the MTR *Ubbo*, the more they thought that it would be easy to use. H5 was validated. However, the need for competence as an APA teacher did not significantly predict perceived ease of use (*p* = 0.872). So, APA teachers’ concern about the enhancement of their teaching skills does not impact significantly the ease of use that they project on Ubbo MTR, and H6 was not validated.

### 3.3 Levels of MTR acceptance and differences between APA students and APA teachers

**Figure 4:**
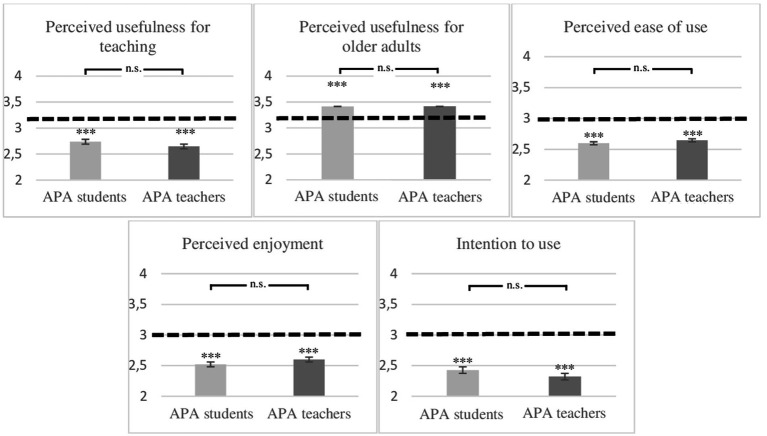
Comparison with the mean of the scale for each variable of the TAM and comparison between APA students and APA teachers. The dotted line represents the mean of the scale. *p < 0.05, **p < 0.01, ***p < 0.001, n.s., non-significant.

The results of the one-sample *t*-tests (presented in Table 2 and Figure 4) showed that the means of perceived usefulness for teaching APA to isolated older adults, perceived enjoyment, and intention to use were significantly lower than the mean of the Likert scale (i.e., 3) for APA teachers and APA students (all *ps* < 0.001). However, one-sample *t-*tests highlighted that the means of perceived usefulness of the MTR for improving older adults’ skills were significantly higher than the mean of the scale for APA students and APA teachers. H7 was validated only for perceived usefulness in improving older adults’ skills. Perceived ease of use was significantly lower than the mean of the Likert scale for APA teachers and APA students (*ps* < 0.001), validating H8. In sum, APA teachers and APA students perceived, before the first use, the MTR *Ubbo* as rather useless for teaching APA to isolated older adults, rather not enjoyable, rather difficult to use, and they rather intend not to use it, but they perceived the MTR useful for improving older adults’ skills.

**Table 2:** Results of one-sample *t*-test for both groups (APA students and APA teachers), means, and standard deviation for each variable of the tested model.

	APA students					APA teachers				
	Mean	Standard deviation	t	*p*	Degrees of freedom	Mean	Standard deviation	t	*p*	Degrees of freedom
Perceived usefulness for teaching	2.738	0.935	40.892	< 0.001	194	2.647	0.946	32.994	< 0.001	138
Perceived usefulness for older adults	3.415	1.003	47.528	< 0.001	194	3.417	1.072	37.571	< 0.001	138
Perceived ease of use	2.597	0.899	40.343	< 0.001	194	2.645	0.942	33.101	< 0.001	138
Perceived enjoyment	2.521	0.967	36.419	< 0.001	194	2.600	0.909	33.714	< 0.001	138
Intention to use	2.427	1.124	30.159	< 0.001	194	2.321	1.189	23.011	< 0.001	138

The results of the *t*-tests for independent samples showed that no significant differences appeared between APA students and APA teachers, regardless of the variable tested (all *ps* > 0.384). Consequently, H9 was not validated.

## 4 Discussion

The aim of this study was to assess MTR acceptance before use by APA teachers (experienced) and APA students (less experienced), for teaching APA remotely to isolated older adults. The present study examined the acceptance degree of this device and assessed psychological variables influencing MTR acceptance by APA teachers and APA students. Studying their acceptance of MTR to teach APA to isolated older adults is an important issue, scarcely addressed in the literature. Indeed, even if older adults intend to use MTR to be supervised in their PA practice, they can access it only if APA teachers also accept to use it.

First, with respect to the validation of the TAM, results showed that the more an APA teacher or an APA student thinks that the MTR is useful for teaching APA to isolated older adults, useful for keeping or improving their physical skills, easy to use, and enjoyable, the more he/she has the intention to use MTR. These results supported the hypotheses tested in this study and were consistent with other findings reported in the literature. Indeed, the TAM has been previously validated with the MTR used in a school context (50), and it has been used with videoconference for tele-neurorehabilitation (51). The present study had to these findings by showing that the TAM explains the acceptance of an MTR to teach remotely APA to isolated older adults by APA teachers and APA students. In addition, in the present study, we tested the robot self-efficacy in the TAM and we showed that the higher the participant’s robot self-efficacy, the more he/she thinks that MTR is easy to use. This relation is not surprising as it can be predicted that if someone feels able to use a device, it will be perceived an easy to use against resources that he/she thinks to have (28, 31). However, the relation between participants’ need for competence in their APA teacher job and perceived ease of use was not significant.

APA teachers and APA students perceived MTR as less useful for teaching APA to isolated older adults, less easy to use, less enjoyable, and had less intention to use it than the mean of the scale. This result is interesting and, to some extent, surprising as, on the one hand, MTR was rather well accepted by older adults before and after use [as supported by previous studies in our group, (15, 36)], while, on the other hand, it seems to be less accepted before the first use by APA teachers and APA students. These results reveal existing barriers (i.e., negative initial representations of MTR technology) to the generalization of the MTR for remotely teaching APA to isolated older adults. A possible explanation is that, though the goal to allow isolated older adults (e.g., who do not have any teaching currently) to benefit remotely coaching was mentioned in the text presenting MTR, participants may have compared teacher’s intervention through MTR and teacher’s intervention in face to face. In other words, they could fill the questionnaire while thinking of teaching face to face as a reference, instead of comparing it with the absence of intervention. Thus, low acceptance scores could be explained by the concept of resistance to change. Bhattacherjee and Hikmet (52) defined resistance to change as “a generalized opposition to change engendered by the expected adverse consequences of change.” This concept can be identified as “reluctance or un-readiness” that “can be overcome cognitively through participation in change” [(53), p.157]. In education topic, teachers’ resistance to change has been explained by their disagreement with their expectations and the efforts required to learn how to use technology (53). The influence of resistance to change on the TAM variables has been shown in literature. First, Guo et al. (54) found that resistance to change influenced significantly and positively perceived usefulness of mobile health services by older adults. This result means that the higher an older adult’s resistance to change, the less he thinks that the device is useful. Second, a study conducted by Nov and Ye (55) about digital library system acceptance showed a negative significant correlation between resistance to change and the perceived ease of use. Authors identified the possible difference between beliefs about the perceived ease of use of participants depending on whether they have yet used the device or not. MTR being an innovative system, very few individuals have yet to use it. So, in the present study, this difference should not be observed. Regarding perceived enjoyment using MTR for teaching APA to isolated older adults scores, they were also lower than the scale mean. APA teacher is a job based on contact with patients. Thus, these practitioners should be particularly attractive for personal interactions. Yet, MTR is an interface for communication, and it can be perceived as a barrier between individuals. This could explain their low perception of enjoyment using MTR in their job context. Finally, Bhattacherjee and Hikmet (52) identified that resistance to use predicted negatively physicians’ intention to use healthcare information technology. In conclusion, the consensus is that resistance to change negatively influences technology acceptance in a variety of contexts, so this concept could explain the low scores of the TAM variables found in the present study.

However, the result of the present study did not follow the pattern of the previous results. Participants also reported a perceived usefulness of MTR significantly higher than the mean of the scale, when perceived usefulness focuses on maintaining or improving the physical capacities of isolated older adults. In other words, APA teachers and APA students thought that MTR was quite useful for keeping or improving older adults’ physical skills. This result could be explained by the function of MTR to provide PA to isolated older adults. As people age, their physiological system declines, leading to limitations in their physical capacities. AP practice benefits physical abilities and precursors of physical incapacities are known (56). Thus, MTR can be thought to be useful for maintaining or improving older adults’ physical capacities, through PA intermediate. This result could seem inconsistent with the precedent one highlighting that participants found the MTR useless for teaching APA to isolated older adults. However, even if MTR is considered as quite useful for keeping or improving older adults’ physical skills, it also puts APA teachers in a teaching situation they have never seen (e.g., teaching remotely APA through MTR). Before the first use, this original and disruptive situation can lead them to think that MTR is useless to teach APA to isolated older adults as they could be in difficulty for teaching. Yet, in the work ergonomics topic, it is known that a worker, regardless of his job, has always the dual concern to do a good job and to save his energy. In the present study, APA teachers and APA students found MTR useful for keeping or improving older adults’ physical skills (i.e., to do a good job), and it corresponds to a new way of teaching that needs to be learned and that they are currently unfamiliar with.

Another important result reported in the present study is the lack of significant difference between APA teachers and APA students in score of each TAM variable. Indeed, one might expect that APA students would have acceptance scores of MTR before use higher than APA teachers. Indeed, they are more familiar with technology in their daily lives because they learn their job in an area where technologies are developed, and their academic curriculum is based on technology support. However, similar results were found in a pedagogical context, evidencing no differences in technology acceptance between in-service teachers and pre-service teachers (57). This can be explained by professional traineeships required for the students’ obtainment of the APA teacher diploma. These professional experiences allow an awareness of job reality and to live professional situations. In this way, APA students develop similar work habits as APA teachers. This may explain the absence of difference between the results of both groups. MTR being an innovative device, neither APA students nor APA teachers are familiar with this, and they have never used it, so they are in similar conditions facing MTR, which can explain the similar MTR acceptance scores of these two populations.

Finally, the present study only investigated MTR acceptance, before use, of APA teachers and APA students. Examining acceptance before use is the first step of robot acceptance (58). This kind of study is relevant because it allows us to identify any initial resistance that potential users may have before using a specific technology for the first time. However, participants could have misrepresentations about the device as their opinion is only based on a presentation text and pictures. While the present study showed that APA students and APA teachers tend to reject MTR before first use, Gallon (59) found that MTR was quite well accepted to teach in a pedagogical context after using it. Participants reported to have reached the goals as quickly through MTR as in face to face with students.

An important public health preoccupation is the social isolation of older adults, which represents a non-negligible risk factor in health deterioration in this population (60). Technology, and more specifically MTR, is a well-tried solution for attenuating social isolation. Indeed, MTR may improve the feeling of the presence of another person due to the live video connection (61). The literature review of Isabet et al. (62) mentioned that compared to other types of social robots, MTR was more beneficial against the social isolation of older adults. MTR also allows enhancing communication, the interactions and connection between older adults and health professionals, caregivers, or family. Nevertheless, it is important not to use technology as a substitute for physical visits that would be counterproductive. Telepresence technology has a vocation to complete the existing organization, not to replace the visits when they are possible.

### 4.1 Limitations and perspectives

Several limitations can be mentioned. First, participants included 37 APA teachers and APA students who had never taught APA to older adults. They may not have identified the specific constraints associated with this type of public and therefore the abilities of the MTR to meet (or not) these needs. Nevertheless, each participant’s APA teaching frequency to older adults was collected, allowing control of this variable. Second, by definition, none of the participants had ever taught APA to isolated older adults, so they may have imagined the MTR use in their usual situation, forgetting that the MTR is destined for an isolated population without any access to an APA teacher physically present. Third, a survey was available online. Even if the conditions of administration of the questionnaire were controlled when the researcher approached the participants directly, it was not when they were contacted via e-mail, so it was impossible to ensure the concentration and attention of participants when they completed the survey. Nevertheless, the survey was available online in order to access a higher number of participants, coming from all of the country, allowing a higher statistical power and the opportunity to conduct structural equation modeling analyses.

The results of the present study allowed to envisage adaptations of the MTR so that it would be better accepted by its potential users. First, results showed that perceived enjoyment influenced intention to use. This trait depends not only on the playfulness of MTR, but also on the technical proper functioning. Thus, the inherent interactive characteristic of mobile telepresence is an important factor for its perceived enjoyment. It is also relevant to ensure the absence of dysfunction, related to a network failure, or a system problem. Then, considering the effect of perceived ease of use on intention to use, and the manifested resistance to change that APA teachers demonstrated, MTR should appear minimalist, and approach a known device that they are already able to use. As Ubbo MTR is just a tablet mounted on a motor basis, it can be complex to make this device more minimalist, and tablet is a commonly known system, so the way in which MTR will be presented to its potential user may be important for the perceived ease of use.

Therefore, it would be interesting to conduct another study investigating the evolution of APA teachers’ and APA students’ acceptance of the MTR after effectively using it, which could enable the participants to identify its interest and to potentially change their initial perceptions, based on a concrete experience with the MTR.

## 5 Conclusion

APA teachers and APA students report a similar MTR acceptance for teaching remotely APA to isolated older adults, before using it for the first time. Specifically, they seem to not perceive immediately its interest in their job and seem to tend to reject it before a first use. However, they think that the MTR would be interesting for keeping or improving isolated older adults’ physical skills, which may sound paradoxical and might suggest that acceptance of the robot might evolve after actual use, but this remains to be investigated. Moreover, this study allowed to understand psychological variables that determine APA teachers’ and APA students’ intention to use, identifying that robot self-efficacy was the most relevant variable to influence acceptance of the MTR. In conclusion, acceptance of the MTR by APA teachers is a decisive criterion for its future use in teaching APA to isolated older adults, while older adults were willing to use this MTR (15, 36). This can contribute to maintaining health of isolated older adults that usually have not access to APA teachers, which is a public health issue. They might improve their quality of life through maintaining their physical skills as long as possible, the aim being to slow down the decline due to aging.

## Data availability statement

The raw data supporting the conclusions of this article will be made available by the authors, without undue reservation.

## Ethics statement

The National Ethics Committee approved the present study (ref. IRB00012476-2023-25-05-253). Written informed consent from the patients/participants or the patients’/participants’ legal guardian/next of kin was not required to participate in this study in accordance with the national legislation and the institutional requirements. Written informed consent was obtained from the individual(s) for the publication of any potentially identifiable images or data included in the supplementary video.

## Author contributions

EN: Data curation, Investigation, Methodology, Validation, Visualization, Writing – original draft. J-JT: Conceptualization, Funding acquisition, Methodology, Supervision, Validation, Writing – review & editing. NM: Conceptualization, Formal analysis, Funding acquisition, Methodology, Project administration, Supervision, Validation, Writing – review & editing.

## Footnotes

^1^https://agebold.com

^2^https://resterjeune.com
