# Supplementary material for: Acceptance of a mobile telepresence robot used to teach adapted physical activity to isolated older adults: extending and testing the technology acceptance model
Source: Front Public Health. 2024 Jul 9;12:1405231. doi: 10.3389/fpubh.2024.1405231 (PMC11264448; doi:10.3389/fpubh.2024.1405231)
Supplement: Supplementary file 1 [file Data_Sheet_1.PDF]

Teaching Adapted Physical Activity to Isolated Older Adults through Mobile Telepresence Robot: Extending and Testing the Technology Acceptance Model

Elodie Navarro, Jean-Jacques Temprado, Nicolas Mascret

Aix Marseille Univ, CNRS, ISM, Marseille, France

Appendix A. Form used in the present study.

Robot de télé-présence mobile :
Activité physique adaptée à distance

IMPORTANT : Pour participer à cette étude, vous devez être enseignant(e) en activité physique adaptée ou en formation pour le devenir. Remplir ce questionnaire prend environ 5 minutes.

Nous vous remercions pour votre participation à cette étude qui porte sur l'usage d'un **robot de télé-présence mobile** (voir photo ci-dessous). Il s'agit d'un système de **visioconférence** mesurant 1.60 m, qui permet de se déplacer dans l'espace, d'entendre (grâce à un micro), de voir (grâce à des caméras), d'être vu (grâce à une tablette tactile) et d'être entendu (grâce à des haut-parleurs) en **temps réel**, ce qui donne l'impression d'être physiquement dans un lieu distant. Ce système a la possibilité d'avancer, de reculer, ou encore de tourner, en toute sécurité grâce à ses capteurs évitant les collisions. Le visage du pilote est diffusé en temps réel sur la « tête » du robot, composée d'un écran tactile et d'une caméra, qui peut pivoter de haut en bas et de droite à gauche, afin d'ajuster le point de vue. Ce dispositif se pilote **à distance** (même à plusieurs milliers de kilomètres) grâce à un réseau Wi-Fi ou 4G, à partir d'une interface sur ordinateur, tablette ou smartphone.

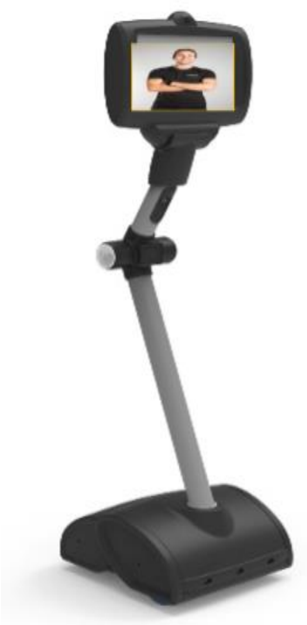

Le robot de télé-présence mobile peut permettre l'**enseignement de l'activité physique adaptée** (APA) à distance à des **séniors isolés**. En effet, une partie de cette population n'a pas forcément la possibilité de se déplacer, se trouve dans des zones reculées ou doit éviter les contacts humains directs pour des raisons médicales, ce qui rend parfois impossible la pratique d'activité physique supervisée en présentiel par un professionnel. Le robot de télé-présence mobile peut donc permettre à ce type de séniors de quand même suivre des séances d'APA, **encadrées** à distance par un enseignant spécialisé en APA, ce qui aurait probablement été impossible sans ce type de dispositif. Il n'est bien entendu pas destiné à remplacer les enseignants en APA (puisqu'ils sont indispensables au fonctionnement du robot, qui doit être piloté par un être humain), il est plutôt destiné à élargir les modalités d'action des enseignants en APA dans les zones dans lesquelles ils ne peuvent pas forcément se déplacer et/ou avec des séniors pour lesquels le contact direct doit être évité pour des raisons médicales.

<u>Même si vous ne l'avez pas encore essayé</u>, nous souhaitons recueillir <u>votre avis</u> concernant ce robot de télé-présence mobile pour **enseigner de l'APA à distance à des séniors isolés**. Pour cela, nous allons vous poser quelques questions dans la suite du document.

Lisez attentivement chaque question et cochez votre réponse. Sachez qu'aucune réponse n'est juste ou fausse, elle doit être personnelle. **<u>C'est votre avis qui est important</u>**. Il est tout à fait normal d'avoir parfois l'impression de répondre plusieurs fois à la même question.

<u>QUESTIONNAIRE n°1</u>

Pour chaque affirmation, cochez <u>la case</u> qui indique votre avis, entre « Pas du tout d'accord » et « Tout à fait d'accord » (les cases intermédiaires servent à nuancer vos réponses). Il n'y a ni bonnes, ni mauvaises réponses, c'est votre avis qui est attendu.

**1**	**Je pense que le robot de télé-présence mobile serait utile pour enseigner de l'APA à distance à des séniors isolés.**
	Pas du tout d'accord □ □ □ □ □ Tout à fait d'accord

**2**	**Je pense que l'utilisation du robot de télé-présence mobile serait simple pour enseigner de l'APA à distance à des séniors isolés.**
	Pas du tout d'accord □ □ □ □ □ Tout à fait d'accord

**3**	**Utiliser le robot de télé-présence mobile pour enseigner de l'APA à distance à des séniors isolés serait amusant.**
	Pas du tout d'accord □ □ □ □ □ Tout à fait d'accord

**4**	**Je pense qu'enseigner de l'APA avec le robot de télé-présence mobile serait utile pour maintenir ou améliorer les capacités physiques des séniors isolés.**
	Pas du tout d'accord □ □ □ □ □ Tout à fait d'accord

**5**	**Si j'avais la possibilité d'avoir facilement accès à ce robot de télé-présence mobile, je l'utiliserais probablement pour enseigner de l'APA à distance à des séniors isolés.**
	Pas du tout d'accord □ □ □ □ □ Tout à fait d'accord

**6**	**Utiliser le robot de télé-présence mobile permettrait d'enseigner efficacement de l'APA à distance à des séniors isolés.**
	Pas du tout d'accord □ □ □ □ □ Tout à fait d'accord

**7**	**Je pense qu'il serait facile d'apprendre à utiliser le robot de télé-présence mobile pour enseigner de l'APA à distance à des séniors isolés.**
	Pas du tout d'accord □ □ □ □ □ Tout à fait d'accord

**8**	**Utiliser le robot de télé-présence mobile pour enseigner de l'APA à distance à des séniors isolés serait agréable.**
	Pas du tout d'accord □ □ □ □ □ Tout à fait d'accord

9	**Je pense qu'enseigner de l'APA avec le robot de télé-présence mobile permettrait aux séniors isolés de maintenir ou d'augmenter leurs capacités physiques.**
	Pas du tout d'accord □ □ □ □ □ Tout à fait d'accord

10	**Si j'avais la possibilité d'avoir facilement accès à ce robot de télé-présence mobile, je souhaiterais l'utiliser pour enseigner de l'APA à distance à des séniors isolés.**
	Pas du tout d'accord □ □ □ □ □ Tout à fait d'accord

11	**Je pense qu'utiliser le robot de télé-présence mobile serait un moyen performant pour enseigner de l'APA à distance à des séniors isolés.**
	Pas du tout d'accord □ □ □ □ □ Tout à fait d'accord

12	**Je pense qu'apprendre à utiliser le robot de télé-présence mobile serait facilement compréhensible pour enseigner de l'APA à distance à des séniors isolés.**
	Pas du tout d'accord □ □ □ □ □ Tout à fait d'accord

13	**Utiliser le robot de télé-présence mobile pour enseigner de l'APA à distance à des séniors isolés serait ludique.**
	Pas du tout d'accord □ □ □ □ □ Tout à fait d'accord

14	**Utiliser le robot de télé-présence mobile pour enseigner de l'APA permettrait de maintenir ou de faire progresser les capacités physiques des séniors isolés.**
	Pas du tout d'accord □ □ □ □ □ Tout à fait d'accord

15	**Si j'avais la possibilité d'avoir facilement accès à ce robot de télé-présence mobile, j'aurais l'intention de l'utiliser pour enseigner de l'APA à distance à des séniors isolés.**
	Pas du tout d'accord □ □ □ □ □ Tout à fait d'accord

QUESTIONNAIRE n°2

Ce questionnaire porte sur votre opinion à propos du robot de télé-présence mobile. Pour chaque affirmation, cochez la case qui indique votre avis, entre « Pas du tout d'accord » et « Tout à fait d'accord » (les cases intermédiaires servent à nuancer vos réponses).

1	**J'ai confiance dans ma capacité à utiliser le robot de télé-présence mobile.**
	Pas du tout d'accord ◻ ◻ ◻ ◻ ◻ Tout à fait d'accord

2	**Je m'attends à être compétent(e) lors de l'utilisation du robot de télé-présence mobile.**
	Pas du tout d'accord ◻ ◻ ◻ ◻ ◻ Tout à fait d'accord

3	**Je suis capable d'utiliser le robot de télé-présence mobile même s'il n'y a personne pour me montrer comment faire.**
	Pas du tout d'accord ◻ ◻ ◻ ◻ ◻ Tout à fait d'accord

QUESTIONNAIRE n°3

Ce questionnaire porte sur votre activité d'enseignant(e) en APA. Pour chaque affirmation, cochez la case qui indique votre avis, entre « Pas du tout d'accord » et « Tout à fait d'accord » (les cases intermédiaires servent à nuancer vos réponses).

1	**Dans mon métier d'enseignant(e) en APA, je maîtrise réellement les tâches à réaliser.**
	Pas du tout d'accord ◻ ◻ ◻ ◻ ◻ Tout à fait d'accord

2	**Dans mon métier d'enseignant(e) en APA, je me sens compétent(e).**
	Pas du tout d'accord ◻ ◻ ◻ ◻ ◻ Tout à fait d'accord

3	**Dans mon métier d'enseignant(e) en APA, je suis bon(ne) dans ce que je fais.**
	Pas du tout d'accord ◻ ◻ ◻ ◻ ◻ Tout à fait d'accord

4	**Dans mon métier d'enseignant(e) en APA, j'ai le sentiment que je peux accomplir même les tâches les plus difficiles.**
	Pas du tout d'accord ◻ ◻ ◻ ◻ ◻ Tout à fait d'accord
